# Identifying pathogenic variants related to systemic lupus erythematosus by integrating genomic databases and a bioinformatic approach

**DOI:** 10.5808/gi.23002

**Published:** 2023-09-27

**Authors:** Ratih Dewi Yudhani, Dyonisa Nasirochmi Pakha, Suyatmi Suyatmi, Lalu Muhammad Irham

**Affiliations:** 1 Department of Pharmacology, Faculty of Medicine, Universitas Sebelas Maret, Surakarta 57126, Indonesia; 2 Department of Histology, Faculty of Medicine, Universitas Sebelas Maret, Surakarta 57126, Indonesia; 3 Faculty of Pharmacy, Universitas Ahmad Dahlan, Yogyakarta 55166, Indonesia

**Keywords:** bioinformatic, genetic factors, genomic variants, single-nucleotide polymorphism, systemic lupus erythematosus

## Abstract

Systemic lupus erythematosus (SLE) is an inflammatory-autoimmune disease with a complex multi-organ pathogenesis, and it is known to be associated with significant morbidity and mortality. Various genetic, immunological, endocrine, and environmental factors contribute to SLE. Genomic variants have been identified as potential contributors to SLE susceptibility across multiple continents. However, the specific pathogenic variants that drive SLE remain largely undefined. In this study, we sought to identify these pathogenic variants across various continents using genomic and bioinformatic-based methodologies. We found that the variants rs35677470, rs34536443, rs17849502, and rs13306575 are likely damaging in SLE. Furthermore, these four variants appear to affect the gene expression of *NCF2*, *TYK2*, and *DNASE1L3* in whole blood tissue. Our findings suggest that these genomic variants warrant further research for validation in functional studies and clinical trials involving SLE patients. We conclude that the integration of genomic and bioinformatic-based databases could enhance our understanding of disease susceptibility, including that of SLE.

## Introduction

Systemic lupus erythematosus (SLE) is a chronic inflammatory-autoimmune disease with a complex multi-organ pathogenesis. The incidence of SLE is highest in women of reproductive age, with a female-to-male ratio around 9:1. The clinical signs of SLE are autoantibody secretion, complement activation, and immune complex deposition, all of which can result in extensive tissue and organ damage and a poor prognosis [1]. The pathogenesis of SLE has yet to be fully understood, but it is believed to involve a complex interplay between genetic predisposition and environmental factors [1,2].

This disease remains a significant challenge for researchers and clinicians due to its complex etiology and pathogenesis, heterogeneous clinical manifestations, and unpredictable exacerbations. Therefore, efforts for identifying potential biomarkers are urgently pursued for several critical reasons. For instance, SLE is often misdiagnosed because no single test is sufficiently sensitive or specific. Additionally, there is no reliable laboratory test for predicting flares and exacerbations, or for identifying specific organ involvement in individual patients with diverse SLE manifestations. Furthermore, the limited availability of biomarkers impedes the discovery of new candidates for SLE [3]. Biomarkers reflect a range of biological processes, including genomic, molecular, histological, and serological markers, that correlate with clinical manifestations or disease pathogenesis [4].

Both twin and familial studies indicate a strong relationship between genetic factors and SLE. Furthermore, over 80 SLE susceptibility loci have been identified through numerous genome-wide and candidate-gene association studies (GWAS) [1]. Through GWAS, it has been documented that several single-nucleotide polymorphisms (SNPs) are associated with the pathogenesis of SLE. However, the disease is not influenced by a single genetic susceptibility alone. Instead, it results from a complex interplay of multiple variants, which could serve as genetic biomarkers. Despite this, the identification of SNPs with missense mutations as the most significant pathogenic variants contributing to SLE remains limited. Consequently, this study aimed to identify genetic variations associated with SLE pathogenesis in a wide range of populations, using genomic databases and bioinformatics approaches. This research is anticipated to provide a comprehensive overview of potential biomarker candidates. These candidates could be used to further investigate the intricate pathogenesis of SLE related to genetic profiles, thereby supporting current targets in SLE drug discovery and development.

## Methods

Identifying genomic variants is crucial not only for understanding the structure of the human genome, but also for gaining a deeper understanding of disease biology. In this study, we utilized several bioinformatic-based approaches to integrate variants associated with SLE. The study was conducted using the National Human Genome Research Institute (NHGRI) GWAS Catalog Database (https://www.ebi.ac.uk/gwas/) with the term "systemic lupus erythematosus" (EFO ID: EFO_0002690), which was downloaded on October 28, 2022 [5]. This yielded 1,259 variants and risk alleles. We then focused on the missense variant due to its potential to alter protein function. A p-value threshold of < 10^-8^ was established to distinguish true positives from false-positives [6,7]. This resulted in a total of 46 variants. Subsequently, an odds ratio greater than one was used to rank the risk of genes affecting SLE, yielding 19 data points after removing duplicates. The SNPnexus (https://www.snp-nexus.org) with PolyPhen-2 databases was employed to determine which variants influenced protein changes in the disease. These were classified as benign, possibly damaging, or probably damaging [8-12], and were extracted on October 28, 2022. A total of four SNPs with 29 variants were predicted as possibly or probably damaging. Population data for the variants were also extracted from SNPnexus using 1000 Genomes Data. The expression profile of the three involved genes was then evaluated using the GTEx Portal (http://www.gtexportal.org/home/) to understand gene expression across various tissues. This data was obtained from the GTEx Portal on October 28, 2022.

**Fig. 1.**
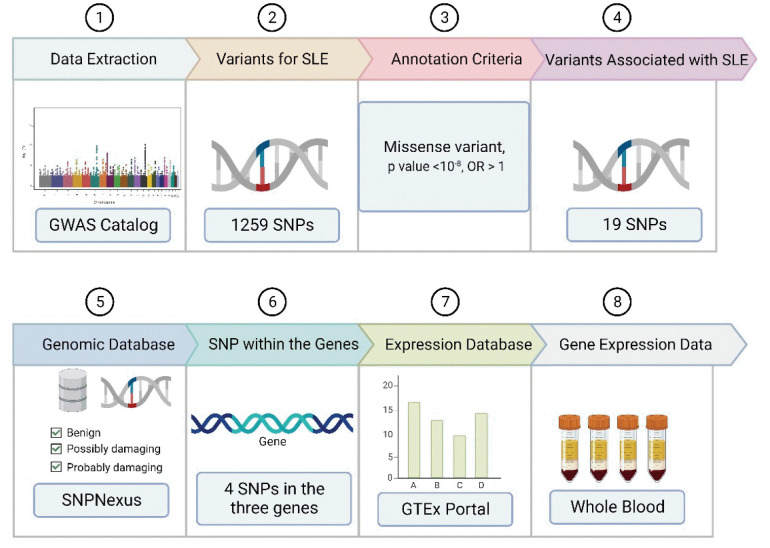
Schematic of bioinformatics pipeline to identify systemic lupus erythematosus (SLE) susceptibility genes across multiple continents. GWAS, genome-wide association studies; SNP, single-nucleotide polymorphism.

A summary of the methodology used to screen for SLE-associated variants is illustrated in the various steps of the bioinformatics pipeline (Fig. 1). A similar method was used by Puspitaningrum et al. [13] to distinguish genomic variants in Sjögren syndrome. Additionally, the same approach was used to identify pathogenic variants of genes related to coronavirus disease 2019 and chickenpox [14].

## Results

### Identification of SLE-associated SNPs

Nineteen SLE-associated SNPs were collected from the GWAS Catalog Database (Table 1). Furthermore, as shown in Table 2, four variant SNPs were identified from the PolyPhen-2 database as contributing to protein-level damage. These SNPs represented three distinct genes: *NCF2*, *TYK2*, and *DNASE1L3*. The *NCF2* gene with the SNP rs13306575 was predicted having possibly damaging by increasing the possibility of having SLE, while the SNP rs17849502 in this gene was predicted to be probably damaging at the protein level. Moreover, both the *TYK2* and *DNASE1L3* genes were predicted to alter the pathogenesis of SLE through a probably damaging mechanism. The strongest contribution was found for the *TYK2* and *DNASE1L3* genes, with the SNPs rs34536443 and SNP rs35677470, respectively, which were predicted to be probably damaging (score 0.999).

### Distribution of SLE-associated SNPs

Table 3 displays the distribution of allele frequencies gathered from the 1000 Genome Database. As indicated in Table 3, this study revealed that, with the exception of rs13306575, Africans and East Asians had allele frequencies of less than 1% or none at all for all variants. In contrast, the South Asian population exhibited an allele frequency of 1%–2% for three SNPs. The American population had frequencies exceeding 2% for all variants. For rs13306575, the highest distribution was observed in East Asia, nearing 7%, while the other SNPs were predominantly distributed in Europe, with frequencies ranging from approximately 3% to 6%.

### Identification of the tissue gene expression of SLE-associated SNPs

**Fig. 2.**
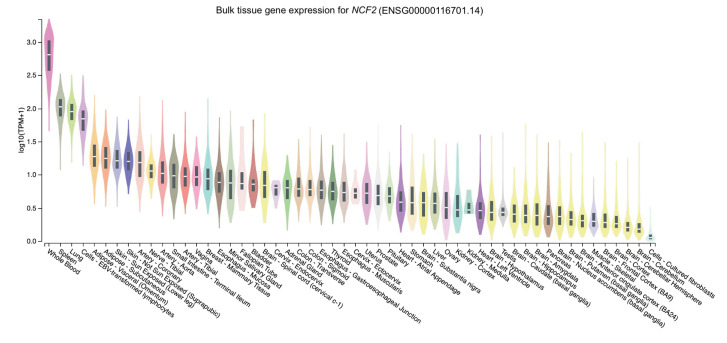
The tissue gene expression for neutrophil cytosolic factor 2 (*NCF2*) according to the database GTEx Portal. TPM, transcripts per million.

**Fig. 3.**
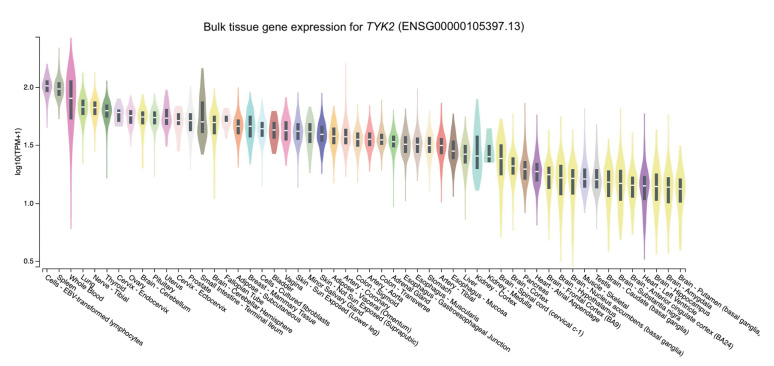
The tissue gene expression for tyrosine kinase 2 (*TYK2*) according to the database GTEx Portal. TPM, transcripts per million.

**Fig. 4.**
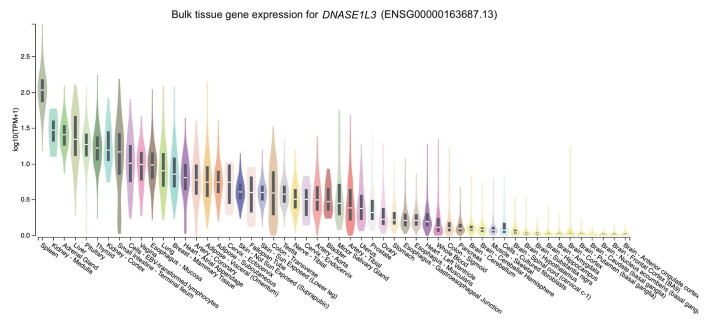
The tissue gene expression for *DNASE1L3* according to the database GTEx Portal. TPM, transcripts per million.

The distribution of the expression of the three genes in different tissues was obtained through GTEx Portal. Of the three genes, *NCF2* was predominantly expressed in whole blood, followed by spleen, lung, and cells (namely, Epstein-Barr virus [EBV]–transformed lymphocytes) (Fig. 2). Similarly, *TYK2* was mainly expressed in cells (EBV-transformed lymphocytes) and spleen, followed by whole blood and the lung (Fig. 3). Meanwhile, the expression of *DNASE1L3* was primarily found in the spleen. In contrast, this gene had low levels of expression in the lung and whole blood (Fig. 4).

## Discussion

### Genetic variants of SLE-associated SNPs

#### *NCF2* gene

Tables 1 and 2 indicate that neutrophil cytosolic factor 2 (*NCF2*), which is located on chromosome 1 and had the SNPs rs17849502 and rs13306575, was one of the strongest contributors to the risk of SLE and could be potentially damaging (score: 0.998). NCF2 is a subunit of the enzyme complex nicotinamide adenine dinucleotide phosphate (NADPH) oxidase, which generates superoxide in phagocytes, including neutrophils and leukocytes. Thus, this enzyme will digest (neutralize) foreign pathogens or remove cell debris [15]. The leukocyte NADPH oxidation complex consists of two membrane proteins (CYBA and CYBB) and three soluble proteins (NCF1, NCF2, and NCF4) alongside a small GTPase (Rac1/2) as an activator [16]. Deficiencies in any of these proteins could lead to the onset of chronic granulomatous disease (CGD), a primary immunodeficiency disorder that typically presents in early childhood. This disease is characterized by low levels of reactive oxygen species (ROS) in the phagosome, which are associated with a deficiency in NADPH oxidase activity. The reduction in NADPH activity leads to abnormal phagocyte digestion and the formation of granulomas in various organs, resulting in recurrent infections, inflammatory disorders, and autoimmunity [17].

In addition to CGD, many studies have identified that alterations in the *NCF2* gene sequence might be related to the onset of lupus and lupus-like diseases [18]. Most autoimmune diseases are characterized by a reduced immunological tolerance mechanism, which leads to the production of autoantibodies and chronic inflammation, causing damage to various tissues and organs. This pathogenic interaction could potentially explain the link between certain autoimmune diseases and reduced ROS production, which is a result of inadequate activity of the NADPH oxidase complex [19].

The rs17849502 SNP is one of the missense variants of the *NCF2* gene that results in a histidine to glutamine (H389Q) substitution in the NCF2 protein. This substitution leads to a reduction of NCF2's binding affinity with the Vav1 protein. These processes further inhibit NADPH oxidase activity due to the stimulation of the signaling pathway involved in Vav1 and could be a genetic risk factor for both adult- and juvenile-onset SLE [20]. Both rs17849502 (*NCF2* gene, substitution His389Gln) and rs13306575 (*NCF2* gene, substitution Arg395Trp) were strongly associated with SLE in North Americans of Hispanic descent (p = 4.91 × 10^-9^ and 1.50 × 10^-11^, respectively). However, only rs17849502 was significantly associated with SLE in North Americans of European descent (EA) (p = 9.47 × 10^-14^). Arg-395 plays a role in stabilizing the interaction between NCF2 and the C-terminal tail of NCF4 via hydrogen bond formation with the carboxyl oxygen of NCF4 residue 339. The Arg-395 → Trp mutation (rs13306575) deteriorates this interaction, which, consequently, will destabilize loop 395–402 of *NCF2* and disrupt the NCF2/NCF4 interaction, followed by disturbance of the NCF2/NCF4/VAV1/RAC1 complex interaction [21].

The connection between a decrease in neutrophil ROS production and the progression of SLE is due to a reduction in the efficiency of efferocytosis and impaired digestion of apoptotic cells. In those genetically predisposed to lupus, this situation could potentially stimulate the production of autoantibodies and chronic inflammation, which are key features of SLE. This could be the primary mechanism explaining the strong correlation between *NCF2* gene polymorphism and SLE risk [15].

There is substantial evidence to suggest that activating NRF2 could be beneficial in the development of SLE. Furthermore, the T cells in SLE patients exhibit a reduced antioxidant capacity, along with decreased levels of NADPH and glutathione. Therefore, strategies aimed at boosting NRF2 activity and reducing intracellular redox metabolism could potentially be effective in managing SLE [22].

#### *TYK2* gene

Tables 1 and 2 show that *TYK2*, which is located on chromosome 19 and had the SNP rs34536443, was also found to be a strong contributor to the risk of SLE and was predicted to be probably damaging (score 0.999). Tyrosine kinase 2 (*TYK2*) resides on chromosome 19q13.2 and encodes a protein belonging to the Janus kinase (JAK) family. TYK2 attaches to the interferon receptor α (IFNAR) on interferon (IFN)-α producing cell surfaces in a state of inactivity. When TYK2 binds to IFNAR, it is phosphorylated and activated during IFN-α exposure. Activated TYK2 phosphorylates IFNAR, resulting in conformational changes that permit the binding of both signal transducer and activator of transcription 3 and 5 (STAT3 and STAT5), mediating cytokine signaling pathways such as interleukin (IL)-12 and IL-23 [23]. It also recruits and phosphorylates STAT1 and 2. STAT1/2 heterodimers then migrate into the nucleus, where they serve as crucial regulators of the expression of several IFN-stimulated genes [24].

Tyrosine kinases are involved in the signaling processes of cells involved in the pathogenesis of autoimmune disease [25]. Moreover, the *TYK2* gene has been identified as a candidate gene linked to autoimmune diseases. Since *TYK2* is on chromosome 19p13.2, part of an SLE linkage region, it has been linked to the pathogenesis of human SLE [23]. The generation of IFN-1 and the regulation of IFN-inducible genes are crucial to the susceptibility, disease activity, and severity of SLE [25]. Numerous studies have considered the IFN-1 pathway in the pathogenesis of SLE. Serum IFN-α levels are increased in patients with SLE; therefore, the secretion of IFN-1 may play a role in the etiology of SLE [26,27].

In addition to its role in the IFN-I and other type I and II cytokine receptor pathways, *TYK2* is involved in other immune systems, such as natural killer cell activity, B and Treg cells' maturation, and the differentiation of Th1 and Th17 cells. Therefore, dysregulation of the expression of *TYK2* has been linked to autoimmune diseases, particularly SLE [24]. *TYK2* polymorphisms have been identified to be associated with SLE [25]. The rare *TYK2* gene variant rs34536443 causes the substitution of a G nucleotide with a C nucleotide, resulting in a Pro1104Ala (P1104A) variant in the *TYK2* protein. This alteration has been hypothesized to promote a conformational change, affecting the folding and function of the *TYK2* protein [27].

Four studies evaluated the association between rs34536443 (g.10352442G>C) SNP and SLE, according to a meta-analysis involving 34 studies on the association of *TYK2* polymorphisms with autoimmune disease. The meta-analysis showed that the rs34536443 C allele protects against SLE (odds ratio [OR], 0.50; 95% confidence interval [CI], 0.50 to 0.57) [24]. The C allele of this SNP might be functional, as it reduces the pSTAT1-induced level of IFN-α in peripheral blood mononuclear cells (PBMCs) relative to cells derived from patients with the G allele, hence decreasing IFNAR signaling [28]. The C allele of rs34536443 diminished p-STAT3 levels induced by IL-23 and IL-12. In addition, it decreased STAT2-induced IFN-β in a murine model of multiple sclerosis (MS) and PBMCs of MS patients carrying the C allele compared to the G allele [29].

In line with that meta-analysis, Diogo et al. [30] documented that allele C of rs34536443 serves to protect against several autoimmune diseases, including inflammatory bowel disease, rheumatoid arthritis, and SLE. Contreras-Cubas et al. [31] demonstrated a protective OR for the C allele of rs34536443 (OR, 0.370; p=0.034) in childhood-onset SLE as an independent predictor after adjusting for sex and ancestry. The protective effect of rs34536443 variants was also found in adult-onset SLE in Mexican patients (OR, 0.277; p=0.008).

The pattern of activated cytokine signaling linked to TYK2 indicated that small molecules that inhibit TYK2 could be potential candidate drugs for SLE patients [32]. A greater understanding of TYK2's molecular and cellular characteristics and studies indicating that *TYK2* gene polymorphisms are protective against developing SLE will lead to the discovery of therapeutic approaches for autoimmune diseases such as SLE.

#### *DNASE1L3* gene

Tables 1 and 2 indicate that the *DNASE1L3* gene, which is located on chromosome 3 and had the SNP rs35677470, had one of the strongest contributions to the risk of SLE and was predicted to be probably damaging (score 0.999). DNASE1L3 is a Mg^2+^Ca^2+^ dependent endonuclease predominantly expressed in myeloid cells [33,34]. One of the roles of DNASE1L3 is to clear DNA released from cells undergoing apoptosis [35]. Meanwhile, it has been found that the absence of DNASE1L3 leads to the accumulation of circulating apoptotic bodies. This triggers an autoimmune response, producing anti-DNA antibodies [34,35]. Yu et al. [36] showed a higher antibody reactivity to DNASE1L3 in SLE patients than in healthy individuals. These anti-DNASE1L3 antibodies are linked significantly with increased SLE disease activity, alongside elevated anti-dsDNA and low complement levels. Moreover, the anti-DNASE1L3 antibody-positive subgroup had higher upregulation of IFN, myeloid/neutrophil/granulocyte, and inflammation modules, indicating that this antibody is associated with increased immune pathway stimulation. However, the data were insufficient to determine the function of anti-DNASE1L3 antibodies in predicting future flares [36].

Moreover, it has been documented that DNASE1L3 deficiency contributes to pediatric-onset SLE, characterized by anti-dsDNA antibodies, reduced complement, and antineutrophil cytoplasmic antibodies [33-35]. Hence, this also suggests that DNASE1L3 might have a protective role from autoimmunity [34]. In addition, *DNASE1L3 –/–* mice developed as a model of pediatric-onset SLE had similar symptoms to humans deficient in DNASE1L3. These mice developed a delay in immune activation, early anti-dsDNA, and anti-chromatin antibodies. The disparity in the timing of the activation of anti-dsDNA and other antibody responses is suggested to be due to the impairment in the initial priming steps, indicating a specific immune activation defect [33].

The missense variant rs35677470 at the *DNASE1L3* locus is linked to the development of SLE [37,38]. The structural analysis by Zervou et al. [37] revealed a modification of the conserved electrostatic network between the guanidinium group of the Arg206 side chain and the carboxylate group of Glu170, forming a strong salt bridge. An electrostatic salt bridge network is formed in conjunction with the Arg208 to Asp219 charge interaction. Meanwhile, the defective rs35677470 SNP allele, with the substitution of arginine by cysteine, disrupts this network and the molecular architecture, including disordered protein folding. This might affect DNASE1L3 enzymatic activity [37]. Additionally, the 3p14.3 locus containing the *DNASE1L3* gene (exon 8) is linked to the risk of SLE, which is a potentially causal missense variant rs35677470 [37,38]. A study by Coke et al. [38] found that the OR for SLE among heterozygous and homozygous carriers of the rs35677470 risk allele was 1.14 (95% CI, 1.05 to 1.24) and 1.68 (95% CI, 1.14 to 2.47), respectively, indicating that both alleles are risk factors. Therefore, SLE patients carrying the rs35677470 risk allele may benefit from DNASE1L3 replacement therapy or other promising therapies to restore DNASE1L3 protein cellular secretion [38]. Furthermore, a previous study found that anti-DNASE1L3 antibodies in SLE were produced by autoreactive VH4-34+ B cells with the 9G4 idiotype, which are intrinsically autoreactive B cells escaping the tolerance checkpoint in SLE. Hence, this previous study indicated that VH4-34+ B cells and 9G4 antibodies are potential targets for SLE treatment [36].

### Distribution of genetic variants of SLE-associated SNPs

This study found that most SNPs were primarily distributed in Europeans, except for rs13306575, which was mainly in East Asia (Table 3). These alleles were also distributed among the American and South Asian populations. This distribution aligns with the documented prevalence of SLE, which is notably higher among Asians and African Americans [39]. A systematic review by Stojan and Petri [40] also showed that the highest estimates of incidence and prevalence of SLE are in North America (23.2/100,000 person-years and 241/100,000 people, respectively). This current study also found that the frequency of alleles of the four SNPs is below 1% in the African population. This finding is supported by a systematic review that found the lowest SLE incidence rates in Africa and Ukraine (0.3/100,000 person-years). Furthermore, Northern Australia has the lowest prevalence of SLE (0 cases among 847 people) [40].

A previous study by Ueki et al. [41] found that rs35677470 (R206C) was accompanied by a homozygous C686 allele in all Asian and African populations, suggesting that the *DNASE1L3* gene exhibits low genetic structure diversity in terms of non-synonymous SNPs since it codes for an enzyme that has been well-conserved throughout human evolution. In contrast, a heterozygous (C686/T686) allele was found in three Caucasian populations (Turkish, German, and Mexican) with a frequency between 3.5% and 15.4%. Compared to Asian and African populations, the genotype distribution of Caucasian populations differed significantly (p = 1.03×10^-17^), suggesting this allele is Caucasian-specific. This variation could be related to the prevalence of autoimmune disease [41].

Furthermore, a previous study by Faezi et al. [27] found that the C allele of rs34536443 in the Iranian population had no impact on SLE susceptibility. This highlighted the importance of genetic divergence in diverse populations and the contribution of different genes to the etiopathogenesis of a multigenic disease like SLE [27]. Meanwhile, the rs34536443 allele was associated with autoimmune disease in the European population. However, the rs34536443 allele is absent or highly uncommon in Asian populations [42].

In addition, numerous studies have indicated that the T allele of the rs17849502 SNP is closely related to the occurrence of adult-onset SLE in various ethnic groups [20,21,43,44]. Furthermore, Bakutenko et al. [15] revealed a strong association between the minor T allele of the rs17849502 SNP and the risk of juvenile-onset SLE in the Belarusian population. The observed frequencies of the T allele in the case groups of juvenile-onset SLE and clinical control were 14.3% and 4.9%, respectively. Moreover, Jacob et al. [20] also observed a significant association of rs17849502 with the European American subgroup for both adult-onset SLE and childhood-onset SLE. However, no association was evident between rs17849502 and SLE in Asian Americans or African Americans [20]. In contrast, Kim-Howard et al. [43] found an association between rs17849502 and SLE in European American, Hispanic, and African American populations. Furthermore, an independent association was observed between a non-synonymous variant rs13306575 and SLE in Hispanic and Korean populations (p_HS_ = 7.04 × 10^−7^ and pKR = 3.30 × 10^−3^, respectively). Additionally, a significant interaction between rs13306575 and rs17849502 was observed in the Hispanic population, significantly elevating the risk (OR, 6.55) [43].

### The tissue gene expression of SLE-associated SNPs

Gene regulatory networks govern both standard and tissue-specific processes, determining gene expression and its levels. While tissue specificity is often described based on gene expression levels, it's understood that individual genes or gene sets alone cannot adequately define the diverse processes that differentiate various tissues. Instead, a combination of regulatory elements, primarily transcription factors, work alongside other genetic and environmental factors to control gene transcription and protein phenotype. The transcriptomic data provided by the Genotype-Tissue Expression (GTEx) consortium presents an unparalleled opportunity to investigate the intricate regulatory patterns required to maintain the specific functional activity of genes across different human tissues [45].

To determine expression levels in human tissues of the genetic variants associated with SLE, we utilized the GTEx Portal database at http://www.gtexportal.org/home/. The database demonstrated that the *NCF2* gene was predominantly expressed in whole blood (Fig. 2). The *TYK2* gene was predominantly expressed in both cells (EBV-transformed lymphocytes) and spleen. In contrast, the expression of *DNASE1L3* was mainly found in the spleen (Figs. 3 and 4).

These *NCF2* and *TYK2* gene expression findings are consistent with the Human Protein Atlas [46,47], indicating that *NCF2* is highly expressed in neutrophils and macrophages. In contrast, the *TYK2* gene is expressed to encode a cytoplasmic and membrane-bound protein in most tissues, particularly the spleen. Slightly different from GTEx, *DNASE1L3* gene expression was highest in the liver, followed by the spleen [48]. In line with the Human Protein Atlas, Chan et al. [49] suggested that DNASE1L3, also known as DNAase γ, belongs to the DNase family. It is predominantly expressed in the liver and lymphoid organs, like the spleen, and is primarily expressed by dendritic cells and macrophages.

The high expression of *NCF2* in neutrophils and macrophages is consistent with previous reports of *NCF2* function. Bakutenko et al. [15] reported that NCF2 is a part of the enzyme NADPH oxidase, which secretes superoxide in neutrophil phagosomes and other phagocytic leukocytes. Superoxide is a crucial component in removing foreign pathogens or cell debris. TYK2 is a member of the Janus kinases, mediating cytokines' intracellular signaling via STAT activation. Although the JAK family of non-receptor kinases is small, this family is an essential intracellular signaling molecule, serving as a crucial connection in the sequence of events from cytokines to cellular responses [50]. This function may explain why TYK2 is predominantly expressed as a cytoplasmic and membrane-bound protein in numerous tissues. The DNASE1L3 protein possesses DNA hydrolysis activity that can cleave single- or double-stranded DNA, which is essential for human plasma DNA homeostasis [51]. A large population of phagocytic cells, including Kupffer cells and macrophages, is found in the liver. Macrophages are essential immune cells that actively play a role in maintaining homeostasis and integrity [52], including DNA integrity. Moreover, the function of DNASE1L3 is DNA hydrolysis and the elimination of apoptotic bodies. This provides a plausible explanation for why *DNASE1L3* is highly expressed in the liver.

GWAS are commonly used to ascertain the statistical relationships between SNPs and numerous significant common diseases, providing fresh insights. Despite the characterization of only a limited number of variants, understanding the functional relationship between these variants and phenotypic traits has proven challenging [53]. This bioinformatics approach has shown that the risk genes identified are linked to SLE pathogenesis. However, given its limitations, additional preclinical (both *in vitro* and *in vivo*) and clinical studies are required to validate and integrate data. This will help to elucidate complex interactions with phenotypes and facilitate the translation of these discoveries into medical practices.

This present bioinformatic study revealed that the genetic variants of the *NCF2*, *TYK2*, and *DNASE1L3* genes were associated with the risk of SLE development in individuals and populations. Both *NCF2* and *TYK2* are highly expressed in whole blood, cells (EBV-transformed lymphocytes), and the spleen. Meanwhile, *DNASE1L3* is primarily expressed in the spleen and liver. We found that two variants of the *NCF2* gene (rs17849502 and rs13306575) were strong contributors to the risk of SLE. Contrastingly, the allele C of rs34536443 in the *TYK2* gene protects against several autoimmune diseases, including SLE. As for *DNASE1L3*, the missense variant rs35677470 was identified as a risk factor for SLE. This study also revealed that *NCF2*, *TYK2*, and *DNASE1L3* could be potential targets, which may facilitate the discovery of new therapeutic approaches for SLE because several variants on those genes are strongly associated with the risk of the disease. Further investigations are necessary to identify potential biomarkers for SLE based on the pathogenic variants found in this investigation.

## Figures and Tables

**Table 1.** Missense variants of SLE-associated SNPs with p < 10^-8^

SNP	p-value
rs35677470	5 × 10^-9^
rs2286672	3 × 10^-9^
rs7097397	2 × 10^-9^
rs1131476	1 × 10^-9^
rs10516487	4 × 10^-10^
rs1061502	9 × 10^-11^
rs1801274	6 × 10^-11^
rs11574637	3 × 10^-11^
rs566731348	4 × 10^-12^
rs2476601	3 × 10^-12^
rs2304256	2 × 10^-12^
rs13306575	2 × 10^-14^
rs17849502	3 × 10^-17^
rs2230926	1 × 10^-17^
rs1131665	9 × 10^-21^
rs34536443	2 × 10^-25^
rs9274384	5 × 10^-31^
rs7097397	5 × 10^-48^
rs9274384	6 × 10^-54^

SLE, systemic lupus erythematosus; SNP, single-nucleotide polymorphism.

**Table 2.** SLE-associated SNPs and their effects at the protein level

SNP	Chromosome	Gene	Score	Prediction
rs35677470	chr3	*DNASE1L3*	0.979–0.999	Probably damaging
rs34536443	chr19	*TYK2*	0.973–0.999	Probably damaging
rs17849502	chr1	*NCF2*	0.919–0.998	Probably damaging
rs13306575	chr1	*NCF2*	0.803–0.806	Possibly damaging

SLE, systemic lupus erythematosus; SNP, single-nucleotide polymorphism.

**Table 3.** The distribution of allele frequencies of four SNPs across multiple continents

SNP	Allele		Allele frequency				
	REF allele	ALT allele	African	American	East Asian	European	South Asian
rs35677470	G	A	0.0030	0.0317	None	0.0527	0.0215
rs34536443	G	C	0.0015	0.0202	None	0.0288	0.0061
rs17849502	G	T	None	0.0245	None	0.0596	0.0143
rs13306575	G	A	0.0008	0.0245	0.069400	None	0.0020

SNP, single-nucleotide polymorphism; REF, reference; ALT, alternative.
